# Social cohesion and quality of life in Bandung: A cross sectional study

**DOI:** 10.1371/journal.pone.0258472

**Published:** 2021-10-13

**Authors:** Sekar Ayu Paramita, Chiho Yamazaki, Lukman Hilfi, Deni Kurniadi Sunjaya, Hiroshi Koyama

**Affiliations:** 1 Department of Public Health, Gunma University, Maebashi, Gunma, Japan; 2 Department of Public Health, Universitas Padjadjaran, Bandung, West Java, Indonesia; Karnataka Veterinary Animal and Fisheries Sciences University, INDIA

## Abstract

In Bandung, Indonesia, urban expansion, rapid economic growth, and population increase present enormous challenges to the maintenance of a high quality of life (QOL) for its citizens. Moreover, income distribution in the city has become more unequal, thereby threatening social cohesion. Such situations led us to investigate the states and correlation of social cohesion and QOL in Bandung. In 2018, we conducted a questionnaire survey of social cohesion and QOL using 13 and 18 question items, respectively. We employed the Rasch model analysis to analyze the logit measures of 752 responses. The results revealed that the population of Bandung has high social cohesion and decent QOL. Our findings suggest that in Bandung QOL is significantly correlated with social cohesion, therefore strategies that seek to enhance social cohesion may be beneficial to improve the QOL.

## Introduction

Various terminologies, such as quality of life (QOL), well-being, happiness, and health, are used to indicate how well human lives are doing [1–3]. Although these terms are interchangeable, QOL is the most prominent and widely used theoretical framework for assessing individual characteristics of different life situation [1, 3].

Since the early 1990s, there has been a growing research interest in the relationship between QOL and social cohesion [3–8]. Social cohesion defined as a state of affairs concerning both the vertical and horizontal interactions among members of a society as characterized by a set of attitudes and norms that include trust, a sense of belonging, and the willingness to participate and help, as well as their behavioral manifestations [5]. Social cohesion characterizes the whole community and impacts the entire neighborhood, regardless of an individual’s characteristics [7, 8]. Manifested in policies, social cohesion can have a positive effect on health through the re-allocation of social and health resources. More cohesive societies invest more in public services such as education, social welfare, and health services, thus narrowing health and education inequalities and reducing unequal access to health and education services [6, 9]. These social circumstances are important factors that improve QOL [10]. Income inequality has a negative effect on an individual’s QOL [11–15]; it also negatively impacts social trust, widens gaps in the community structures [12, 14, 16], and reduces contact between the rich and the poor, thereby reducing social cohesion [12].

It is important to recognize that both health promoting and damaging behaviors can spread through close social interactions, depending on the social context [17]. A cohesive society is a crucial societal condition for positive life evaluation and well-being, and people living in cohesive societies have been found to be happier and more satisfied with life and achieve better health status [4, 18–22]. However, a recent review highlights a “dark side” to social cohesion that can promote damaging health behaviors, such as smoking and drinking, and even increase the symptoms of depression [17].

The economy of Bandung, Indonesia, has grown rapidly in recent decades. Its growth rate is the highest in West Java Province, and it is even higher than the national growth rate [23]. Commerce and industry were the main contributors to the Bandung economy until urban expansion shifted the economic policy toward the service sectors, such as tourism. These new conditions have attracted people to relocate and settle permanently in the city, leading to a dramatic population increase. This aspect has created challenges to maintaining a high QOL for the city’s residents [23]. In addition, income distribution in the city has become more unequal [24], which may threaten social cohesion. Given this situation, we set out to investigate the states and correlation of social cohesion and QOL in Bandung. However, there is no such evaluations to date. Understanding social cohesion and the ways in which it relates to QOL may be valuable in policymaking and planning of the local governments and communities. Furthermore, given that social cohesion is a central part of social capital, the study results are of value to not only in public health, but also in other fields such as economy, psychology and sociology.

## Materials and methods

This is a quantitative cross-sectional study. The survey instrument that we developed comprised 8 items on sociodemographic attributes items, 13 items on social cohesion, and 18 items on QOL. Before the administration of the survey, the questionnaire was tested for reliability using responses from randomly selected 166 citizens of Bandung. We followed Rasch analytical procedures to document the measurement properties of the questionnaire (e.g., reliability and construct validity), and obtained acceptable levels of fit to the Rasch expectation (Cronbach’s alpha = 0.70, model RMSE = 0.08, SD = 0.54, item separation = 7.14, and item reliability 0.98) [25].

We included the following sociodemographic attributes: age of the respondent at interview, gender, place of birth, living area, level of education, occupation, marital status, and income per capita. We used dummy variables for each age group (0 = ≤ 35; 1 = 36–55; 2 = ≥56), gender (0 = male; 1 = female), place of birth (0 = not Bandung; 1 = Bandung), living area (0 = Bojonagara; 1 = Tegalega; 2 = Cibeunying; 3 = Kerees; 4 = Gedebage; 5 = Kordon; 6 = Ujungberung; 7 = Arcamanik), level of education (0 = junior high school and below; 1 = completed senior high school; 2 = completed higher education), occupation (0 = unemployed; 1 = student: 2 = taking care of household; 3 = government employee; 4 = private sector employee; 5 = business owner; 6 = military or police force: 7 = other), marital status (0 = not married; 1 = married; 2 = divorced/widower), and income per capita (0 = <1,554,360; 1 = 1,554,360–3,091,344; 2 = 3,091,345–5,000,000; 3 = 5,000,001–10,000,000; 4 = >10,000,000 IDR).

We measured social cohesion using Chan et al.’s social cohesion framework [5], which comprises two dimensions (horizontal versus vertical) and two components (objective versus subjective). The horizontal dimension focuses on the relationships among different individuals and groups within the society while the vertical dimension explores the relationship between the state and its citizens (or civil society) [5]. The subjective component includes trust, sense of belonging willingness to cooperate and help, while the objective component refers to the actual cooperation and participation of the members of the society [5]. The QOL part of the questionnaire consisted of 18 items that based on the United Nations Development Programme’s concept of QOL, which includes happiness and other well-being questions as direct estimates QOL [26–29].

Ethical approval for the study was obtained from the Research Ethical Committee of Universitas Padjadjaran, Indonesia (No.157/UN6.KEP.EC.2018), and a survey permit was obtained from the Health Office of Bandung City (Surat Keterangan No.070/5757-Dinkes). We conducted the survey in all eight subregions of Bandung from May to July 2018. In 2018, the city’s population was 2,440,717 [30]. To ensure that the 99% confidence level estimate for the social cohesion and QOL in Bandung population is within the 5% confidence interval, a minimum sample of size 665 was required. Thus, random sampling stratified by geographic location was employed to ensure that the final sample composition was geographically representative. Respondents were given explanatory statements of the objectives and course of the study, together with the written informed consent before responding to the questionnaire. The survey was facilitated by trained surveyors.

Questionnaire responses to the social cohesion and QOL items were provided in a 7-point Likert scale. The shortcomings of ordinal data include the respondent’s abilities, attitudes, personality traits, and the item difficulty. Therefore, we used the Rasch measurement of Ordered Polytomous Items to convert the responses into values that could be evaluated in a logit form [31, 32]. We followed Rasch analysis procedures to document the measurement properties of the questionnaire (e.g., reliability and construct validity) [31], and reached acceptable levels of fit to the Rasch expectation. The Rasch analysis is also a useful post-hoc analytic approach in determining the optimal categorization of an ordered-response scale [33]. Rasch analysis was conducted using Winsteps 3.75.

In the questionnaire, the possible logit ranges of social cohesion measures were from –3.51 to 3.91, while the possible logit ranges of QOL measures were from –4.23 to 6.03. We simply divided these ranges into three equal intervals, and therefore, the levels of social cohesion and QOL can be interpreted as low, medium, and high. Descriptive statistics were calculated for social cohesion and QOL, and they were presented as the mean, minimum, and maximum. To further determine whether social cohesion and QOL differ among the sociodemographic groups, mean scores were compared across sociodemographic groups using Analysis of Variance (ANOVA) followed by Tukey’s post hoc test, and a p value < 0.05 level was considered statistically significant. Pearson correlation coefficient was employed to examine the correlation between social cohesion and QOL. Statistical analysis was conducted using EZR [34].

## Results and discussion

### Respondent characteristics

We retrieved 752 responses. Respondents possessed varied sociodemographic attributes, as presented in Table 1. The mean age was 37 years with a standard deviation of 14, and 57.2% of the respondents were male. There were 459 respondents (61%) who were born in Bandung. Large portion of respondents (181, 24%) lived in Cibeunying area. Regarding level of education, 528 respondents (70.2%) reported completing higher education. In total, 253 respondents (33.6%) work in the private sector. Most of respondents were married (470, 62.5%). Only 737 respondents disclosed their income. Indonesian law requires the amount of the minimum wage to be fixed with reference to a decent standard of living [35]. The provincial minimum wage at the time of the survey was 1,554,360 IDR per month. The income per capita of 472 respondents (62.8%) were lower than the provincial minimum wage. The city’s minimum wage at the time of the survey was 3,091,344 IDR per month, and 634 respondents (84.3%) had lower income per capita compared with this minimum. The income per capita data in our study reflect individual income share in the respondent’s household, so the comparison with minimum wage could illustrate whether the respondents have achieved a decent living standard. Additionally, the disproportionate division of the number of respondents according to income groups demonstrated the level of income inequality in the city.

**Table 1:** Sociodemographic attributes of the respondents.

*Sociodemographic attributes*		*Respondents (n = 752)*	
		*n*	*%*
*Age*	≤ 35 years old	398	52.9%
	36–55 years old	264	35.1%
	≥56 years old	90	12.0%
*Gender*	Male	430	57.2%
	Female	322	42.8%
*Place of birth*	Not Bandung	293	39.0%
	Bandung	459	61.0%
*Living area*	Bojonagara	132	17.6%
	Tegalega	77	10.2%
	Cibeunying	181	24.1%
	Kerees	109	14.5%
	Gedebage	40	5.3%
	Kordon	55	7.3%
	Ujungberung	52	6.9%
	Arcamanik	106	14.1%
*Level of education*	Middle school or less	19	2.5%
	High school	205	27.3%
	Higher education	528	70.2%
*Occupation*	Unemployed	33	4.4%
	Student	95	12.6%
	Taking care of household	55	7.3%
	Government employee	105	14.0%
	Private sector employee	253	33.6%
	Business owner	69	9.2%
	Military or police force	6	0.8%
	Other	136	18.1%
*Marital status*	Single	252	33.5%
	Married	470	62.5%
	Divorce/widower	30	4.0%
*Income percapita (n = 737)*	<1,554,360	472	62.8%
	1,554,360–3,091,344	162	21.5%
	3,091,345–5,000,000	61	8.1%
	5,000,001–10,000,000	29	3.9%
	>10,000,000	13	1.7%

Income per capita was calculated as the average of total family income per month divided by the number of family members; IDR = Indonesian Rupiah.

### States of social cohesion

**Fig 1:**
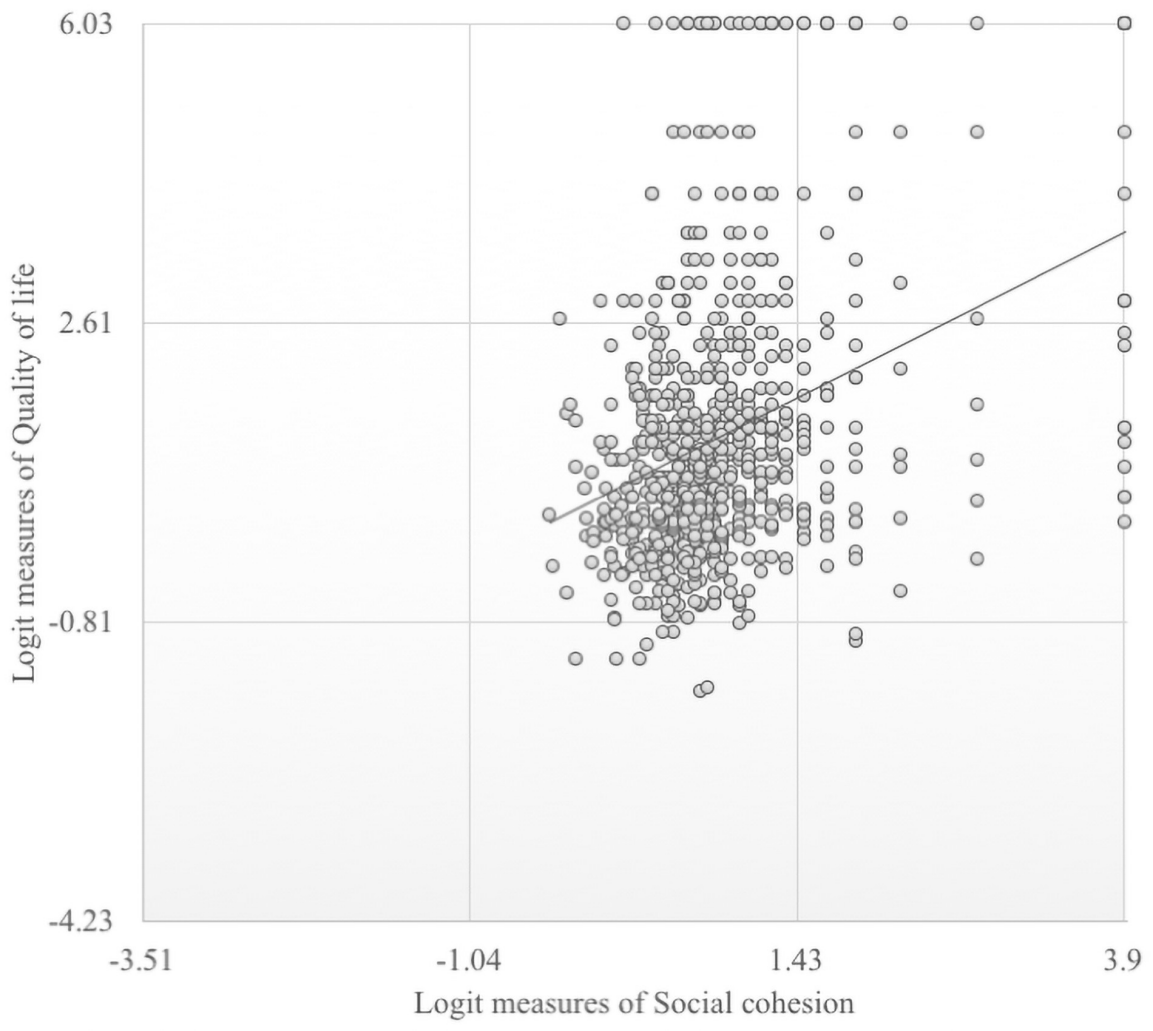
Respondents’ measures of social cohesion and quality of life. This figure describes the distribution of social cohesion and QOL of the respondents in logit measures; On the basis of our instruments, the possible logit ranges of social cohesion measures are from –3.51 to 3.91 while the possible ranges of QOL measures ranges from –4.23 to 6.03. For the social cohesion measures, −3.51 to −1.04 was interpreted as low, −1.04 to 1.43 was interpreted as medium, and 1.43 to 3.9 was interpreted as high. For the QOL measures, −4.23 to −0.81 was interpreted as low, −0.81 to 2.61 was interpreted as medium, and 2.61 to 6.03 was interpreted as high.

Fig 1 exhibits the logit measures of 752 responses, with social cohesion measures ranging from –0.44 to 3.91, and a mean of 0.87 (Fig 1). The possible ranges of social cohesion measures in the questionnaire are from –3.51 to 3.91, and the majority of respondents (n = 656; 87.2%) have medium levels of social cohesion.

Table 2 presents a summary of the results for the social cohesion analysis based on the sociodemographic attributes. The younger respondents had a slightly higher social cohesion mean than older respondents while female respondents had a slightly higher social cohesion measure (0.91) than their males counterparts (0.84). There’s only a slight difference in social cohesion measure from respondents born in Bandung (0.88) and outside of Bandung (0.85). Based on the living area, Kordon has the highest social cohesion measures (0.96), followed by Arcamanik (0.92). According to Bandung city the data profile of Bandung [24], Arcamanik has the largest park area in the city. Other studies have suggested that the availability of urban green spaces provides opportunities for social interactions to take place that may play a vital role in increasing social cohesion [36]. Respondents with educational attainment of middle school or less level have the highest social cohesion mean (0.93), although the maximum measure of this group (2.21) does not reach the highest social cohesion measure (3.91). Respondents occupied in the military or police force had the highest mean (1.46). The social cohesion mean of respondents that we assume spend more time in their neighborhood was found to be higher. These respondents include the unemployed (0.91) and those taking care of household (0.91) and were compared with the respondents who work outside the home such as government employees (0.9), private sector employees (0.86), or business owners (0.76). Married respondents had the highest mean of social cohesion (0.92) compared with single (0.79) or divorced/widower (0.73). The maximum measure for divorced/widower respondents (2.21) did not reach the highest social cohesion measure (3.91). Maximum measure of respondents in highest income group (1.66) did not reach highest social cohesion measure (3.91). We examined correlations for each sociodemographic with social cohesion (Table 2). None of the sociodemographic attributes reveal significant correlation with social cohesion.

**Table 2:** Social cohesion and quality of life analysis based on the sociodemographic attributes.

*Socio-demographic attributes*		Social cohesion				Quality of life				Social cohesion with Quality of life	
		*Mean*	*Min*	*Max*	p-value	*Mean*	*Min*	*Max*	p-value	*Coef*	*p-value*
*All respondents*		0.87	-0.44	3.91		1.34	-1.60	6.03		**0.34**	**<0.001**
*Age*	≤ 35 years old	0.89	-0.36	3.91	0.525	1.40	-1.60	6.03	0.560	**0.36**	**<0.001**
	36–55 years old	0.87	-0.44	3.91		1.27	-0.93	6.03		**0.37**	**<0.001**
	≥56 years old	0.79	-0.15	3.91		1.29	-1.23	6.03		0.07	0.461
*Gender*	Male	0.84	-0.44	3.91	0.161	1.30	-1.60	6.03	0.427	**0.37**	**<0.001**
	Female	0.91	-0.30	3.91		1.39	-1.55	6.03		**0.30**	**<0.001**
*Place of birth*	Not Bandung	0.85	-0.30	3.91	0.659	1.36	-1.23	6.03	0.743	**0.31**	**<0.001**
	Bandung	0.88	-0.44	3.91		1.32	-1.60	6.03		**0.36**	**<0.001**
*Living area*	Bojonagara	0.81	-0.41	3.91	0.758	1.37	-0.93	6.03	0.308	**0.5**	**<0.001**
	Tegalega	0.81	-0.23	3.91		1.09	-1.23	4.80		**0.39**	**<0.001**
	Cibeunying	0.91	-0.28	3.91		1.35	-1.55	6.03		**0.26**	**<0.001**
	Kerees	0.82	-0.12	3.91		1.60	-0.61	6.03		**0.42**	**<0.001**
	Gedebage	0.91	-0.30	3.91		1.64	-0.93	6.03		0.15	0.351
	Kordon	0.96	-0.30	3.91		1.24	-0.48	6.03		**0.54**	**<0.001**
	Ujungberung	0.83	0.06	3.91		1.07	-0.82	6.03		**0.54**	**<0.001**
	Arcamanik	0.92	-0.44	3.91		1.26	-1.60	6.03		0.14	0.140
*Level of education*	Middle school or less	0.93	-0.15	2.21	0.036	1.41	-0.61	6.03	0.877	**0.65**	**0.002**
	High school	0.75	-0.41	3.91		1.34	-0.82	6.03		**0.34**	**<0.001**
	Higher education	0.91	-0.44	3.91		1.33	-1.60	6.03		**0.34**	**<0.001**
*Occupation*	Unemployed	0.91	-0.02	3.91	0.273	1.20	-0.82	4.07	0.870	0.16	0.387
	Student	0.78	-0.02	1.88		1.30	-0.74	6.03		**0.46**	**<0.001**
	Taking care of household	0.91	-0.30	2.80		1.63	-0.95	6.03		0.04	0.764
	Government employee	0.90	-0.30	3.91		1.24	-1.23	6.03		**0.53**	**<0.001**
	Private sector employee	0.86	-0.41	3.91		1.35	-1.55	6.03		**0.37**	**<0.001**
	Business owner	0.76	-0.44	2.80		1.21	-1.60	6.03		0.19	0.122
	Military or police force	1.46	0.50	3.91		1.32	-0.66	2.85		0.80	0.053
	Other	0.93	0.03	3.91		1.39	-0.77	6.03		**0.34**	**<0.001**
*Marital status*	Single	0.79	-0.36	3.91	0.032	1.31	-1.60	6.03	0.924	**0.30**	**<0.001**
	Married	0.92	-0.44	3.91		1.36	-1.55	6.03		**0.37**	**<0.001**
	Divorce/widower	0.73	-0.15	2.21		1.29	-0.37	4.07		0.09	0.644
*Income percapita (n = 737)*	<1,554,360	0.86	-0.41	3.91	0.101	1.31	-1.60	6.03	0.796	**0.36**	**<0.001**
	1,554,360–3,091,344	0.87	-0.44	3.91		1.45	-1.23	6.03		**0.30**	**<0.001**
	3,091,345–5,000,000	0.76	-0.23	3.91		1.23	-0.79	6.03		**0.47**	**<0.001**
	5,000,001–10,000,000	1.20	0.22	3.91		1.41	-0.74	6.03		0.23	0.236
	>10,000,000	0.82	0.43	1.66		1.11	-0.93	4.80		0.12	0.701

Income per capita was calculated as the average of total family income per month divided by the number of family members; IDR = Indonesian Rupiah; Social cohesion and quality of life (QOL) values are displayed in logit measures from Rasch analysis (using Winsteps 3.75); the possible logit ranges of the social cohesion measures = –3.5–3.91; the possible logit ranges of the QOL measures = –4.23–6.03; ^a^Test comparing sociodemographic groups using ANOVA followed by Tukey’s post hoc test (using EZR [34]); ^b^Pearson correlation was conducted to examine correlation between social cohesion and QOL (using EZR [34]); values in **bold** indicate statistical significance (p < 0.05).

### States of QOL

Fig 1 shows the possible logit ranges of QOL measures ranges from –4.23 to 6.03 (Fig 1). Based on the measurements of the 752 respondents, the QOL measure ranges from –1.6 to 6.03, and the mean was 1.34. The majority of respondents (n = 619; 82.3%) are considered to have medium level QOL.

**Fig 2:**
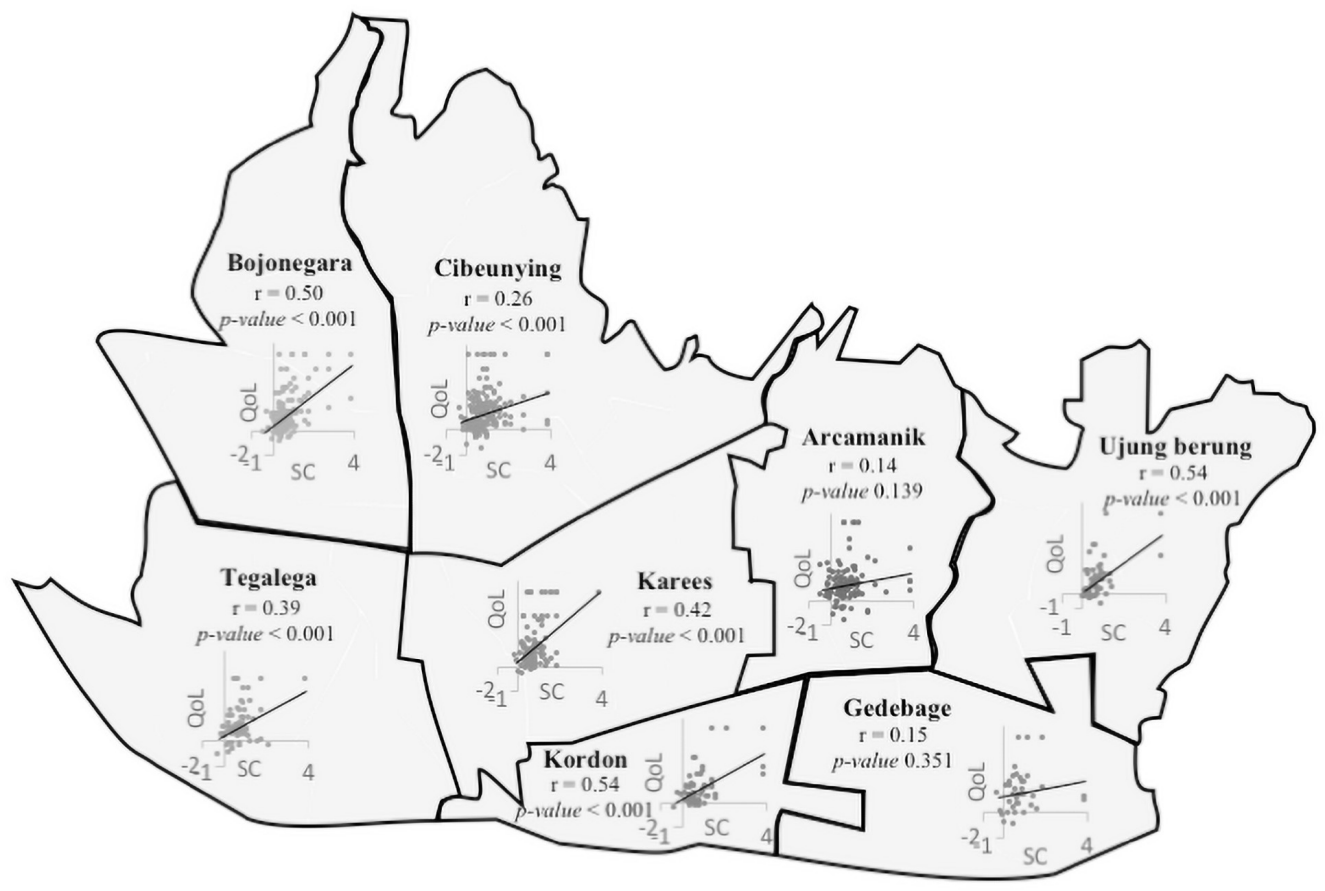
Correlation of social cohesion and quality of life in the eight subregions of Bandung City. SC = social cohesion; QOL = quality of life. This figure was drawn by the first author (SAP) using Microsoft PowerPoint. The figure is only provided for illustrative purposes.

Table 2 presents a summary of the results of QOL analysis based on sociodemographic attributes. Based on the age group, mean of QOL is the highest for respondents aged ≤ 35 years old (1.4). QOL measures based on gender (male = 1.3; female = 1.39) and place of birth (not Bandung = 1.36; Bandung = 1.32) revealed only a slightly difference. Based on the living area (Fig 2), Gedebage had the highest QOL measures (1.64), while Tegalega had the second lowest QOL (1.09) compared with other areas, and the maximum measure for this area (4.8) did not reach maximum QOL measures (6.03). Respondents with educational attainment of middle school or less had the highest mean QOL (1.41). Respondents that were taking care of households had the highest QOL (1.6), while the maximum measures of respondents who were unemployed (4.07) or in the military or police force (2.85) did not reach the maximum QOL measures (6.03). Married respondents had the highest mean QOL (1.36) compared with single (1.31) or divorced/widower (1.29) respondents. The maximum measure of divorced/widower respondents (4.07) did not reach highest QOL measures (6.03). The maximum measure for respondents in the highest income group (4.8) did not reach the highest QOL measures (6.03). We examined the correlations of each sociodemographic category with QOL (Table 2) and found no significant difference in QOL among the sociodemographic groups.

### Correlation between social cohesion and QOL

The Pearson correlation coefficient shows a significant positive correlation between social cohesion and QOL (Table 2). This is consistent with other studies [37–39] which have found that QOL is higher in more cohesive societies. Berger-Schmitt argues that QOL includes several components and aspects that are either a part of the concept of social cohesion or an outcome of social cohesion [3], and this aspect may explain the significant correlation between both the variables.

When respondents are differentiated according to sociodemographic attributes, social cohesion and QOL show significant correlation in all groups according to gender, place of birth and education level.

We found that social cohesion and QOL were significantly correlated in younger age groups (≤ 35 and 36–55 years). Other studies [7, 40] have revealed that the elderly population relies more on their neighborhoods. Therefore, we might presume that the QOL of that demographic also relies on social cohesion. However, our analysis found that correlation of social cohesion and QOL in respondents in the ≥56 years age group did not show significant correlation (Table 2).

In 2011, Bandung government consolidated 30 *Kecamatan* (sub-districts) into eight areas (*Satuan Wilayah Kerja*), namely, Bojonagara, Tegalega, Cibeunying, Kerees, Gedebage, Kordon, Ujungberung, and Arcamanik [41]. The government stated that this arrangement would help optimize development based on the potential of each area and reduce income inequality among areas. The development plans for the Bojonegara and Cibeunying focus on tourism and commerce. The development plan for Gedebage focuses on government offices, urban green spaces, housing, rice fields and commerce, while Tegalega focuses on industrial development. Ujungberung and Arcamanik focus on providing affordable housing. The objective of this consolidation is to reorganize the City to support city planning from 2011 to 2031. We analyzed the states and correlation of social cohesion and QOL for each area, and the results show that there are no significant differences in the states of social cohesion and QOL measures among areas (Table 2). However, social cohesion and QOL were significantly correlated in all areas, with the exception of Gedebage and Arcamanik (Fig 2).

When respondents were differentiated according to occupation, a significant correlation between social cohesion and QOL emerged for students, government and private employees, and “other” occupations. A significant correlation was also found in single or married respondents, but not in divorced/widower respondents.

The respondents were disproportionally distributed according to their income groups. Social cohesion and QOL were significantly correlated in respondents in the lower income groups (≤5,000,000 IDR) but not in the higher income groups (>5,000,000 IDR), as shown in Table 2. It is possible that among the higher income groups, respondents are not reliant on social cohesion to improve their QOL, as higher incomes may yield better living conditions or opportunities considered beneficial to QOL [42].

## Conclusion

Results from this study demonstrate that the population of Bandung has high social cohesion, given that the mean (0.87) falls within the high social cohesion group and none of the respondents are categorized in the low social cohesion group. Results for QOL suggest that the population has a decent QOL, given that the mean (1.34) falls within the medium QOL group. These findings reveal that neither social cohesion nor QOL have a significant correlation with the sociodemographic attributes of the respondents. However, social cohesion is significantly correlated with QOL, especially among younger respondents (≤ 35 and 36–55 years), both male and female, those born in Bandung and outside of Bandung, in all areas except for Gedebage and Arcamanik, at all levels of educational attainment, among students, government and private employees, and those in “other” occupations, among single or married respondents, and those in lower income groups. These results suggest that strategies that seek to enhance social cohesion may also beneficial for improving QOL.

## Supporting information

S1 File Questionnaire. (PDF)

S2 File Data. (XLSX)
