# Supplementary material for: Social cohesion and quality of life in Bandung: A cross sectional study
Source: PLoS One. 2021 Oct 13;16(10):e0258472. doi: 10.1371/journal.pone.0258472 (PMC8513873; doi:10.1371/journal.pone.0258472)
Supplement: S1 File — (PDF) [file pone.0258472.s001.pdf]

KUISIONER PENELITIAN

Instruksi
Lengkapi pertanyaan berikut seakurat mungkin. Informasi Anda akan dijaga kerahasiaannya.

No	Pertanyaan	Jawaban
1	Tanggal lahir (tanggal/bulan/tahun)	…
2	Jenis kelamin	a. Pria b. Wanita
3	Kota kelahiran	…
4	Alamat saat ini (SWK)	…
5	Pendidikan terakhir	a.Tidak ada b.SD/ sederajat c.SMP/ sederajat d.SMA/ sederajat e. D1/D3/D4/S1/S2/S3
6	Pekerjaan	a. Tidak bekerja b. Pelajar c. Mengurus rumah tangga d. PNS<br>e. Pegawai swasta f. Wiraswasta g. TNI/ POLRI h. Lainnya
7	Status pernikahan	a. Belum menikah b. Menikah c. Janda/duda
8	Pendapatan keseluruhan anggota keluarga per bulan dibagi jumlah anggota keluarga yang tinggal satu rumah termasuk Anda (Rp)	a. <1,554,360 b. 1,554,360 - 3,091,344 c. 3,091,345 - 5,000,000<br>d. 5,000,001 - 10,000,000 e. >10,000,000

**Instruksi**<br>Lingkari jawaban yang paling sesuai dengan pendapat Anda. Tidak ada jawaban yang salah.								
**No**	**Pertanyaan**	**Jawaban**						
9	Seberapa kuat rasa memiliki Anda terhadap Kota Bandung? (1= “tidak ada rasa memiliki sama sekali”, 7= “rasa memiliki sangat kuat”)	1	2	3	4	5	6	7
10	"Saya merasa bangga menjadi warga Kota Bandung’’ (1="sangat tidak setuju", 7="sangat setuju")	1	2	3	4	5	6	7
11	‘‘Kota Bandung adalah rumah saya, apapun yang terjadi’’ (1="sangat tidak setuju", 7="sangat setuju")	1	2	3	4	5	6	7
12	Seberapa besarkah rasa percaya anda terhadap Pemerintah Kota Bandung? (1= “tidak percaya sama sekali”, 7= “sangat percaya”)	1	2	3	4	5	6	7
13	‘‘Saya bersedia membayar pajak lebih banyak apabila digunakan untuk memperbaiki kesejahteraan masyarakat’’ (1="sangat tidak setuju", 7="sangat setuju")	1	2	3	4	5	6	7
14	‘‘Saya bersedia menyisihkan waktu luang saya untuk menjadi sukarelawan.’’ (1="sangat tidak setuju", 7="sangat setuju")	1	2	3	4	5	6	7

**Instruksi**<br>Lingkari jawaban yang paling sesuai dengan pendapat Anda. Tidak ada jawaban yang salah.								
**No**	**Pertanyaan**	**Jawaban**						
15	‘‘Saya mempercayai tetangga tetangga saya’’ (1="sangat tidak setuju", 7="sangat setuju")	1	2	3	4	5	6	7
16	Dalam kelas sosial masyarakat, Anda termasuk kedalam kelas sosial yang mana?	Kelas bawah		Kelas menengah kebawah	Kelas menengah		Kelas menengah atas	Kelas atas
17	"Apabila seseorang berasal dari kelas sosial yang lebih rendah, saya akan menjaga jarak dengan orang tersebut." (1="sangat tidak setuju", 7="sangat setuju")	1	2	3	4	5	6	7
18	"Apabila seseorang berasal dari kelas sosial yang lebih tinggi, saya akan menjaga jarak dengan orang tersebut." (1="sangat tidak setuju", 7="sangat setuju")	1	2	3	4	5	6	7
19	"Apabila seseorang mempunyai pandangan politik yang berbeda, saya akan menjaga jarak dengan orang tersebut." (1="sangat tidak setuju", 7="sangat setuju")	1	2	3	4	5	6	7

**Instruksi**<br>Lingkari jawaban yang paling sesuai dengan pendapat Anda. Tidak ada jawaban yang salah.								
**No**	**Pertanyaan**	**Jawaban**						
20	"Apabila seseorang mempunyai pandangan agama yang berbeda, saya akan menjaga jarak dengan orang tersebut." (1="sangat tidak setuju", 7="sangat setuju")	1	2	3	4	5	6	7
21	"Apabila seseorang adalah seorang pendatang, saya akan menjaga jarak dengan orang tersebut." (1="sangat tidak setuju", 7="sangat setuju")	1	2	3	4	5	6	7
22	"Apabila seseorang merupakan penerima tunjangan pemerintah, saya akan menjaga jarak dengan orang tersebut." (1="sangat tidak setuju", 7="sangat setuju")	1	2	3	4	5	6	7
23	Seberapa puaskah Anda terhadap komunitas Anda? (1="sangat tidak puas", 7="sangat puas")	1	2	3	4	5	6	7
24	Seberapa puaskah Anda terhadap kehidupan sosial Anda? (1="sangat tidak puas", 7="sangat puas")	1	2	3	4	5	6	7

**Instruksi**<br>Lingkari jawaban yang paling sesuai dengan pendapat Anda. Tidak ada jawaban yang salah.								
**No**	**Pertanyaan**	**Jawaban**						
25	Seberapa puaskah Anda terhadap keharmonisan dalam keluarga Anda? (1="sangat tidak puas", 7="sangat puas")	1	2	3	4	5	6	7
26	Seberapa puaskah Anda terhadap hubungan personal Anda? (1="sangat tidak puas", 7="sangat puas")	1	2	3	4	5	6	7
27	Seberapa puaskah Anda terhadap kondisi kesehatan Anda? (1="sangat tidak puas", 7="sangat puas")	1	2	3	4	5	6	7
28	Seberapa puaskah Anda terhadap pendidikan dan keterampilan yang Anda miliki? (1="sangat tidak puas", 7="sangat puas")	1	2	3	4	5	6	7
29	Seberapa puaskah Anda dengan pekerjaan Anda? (1="sangat tidak puas", 7="sangat puas")	1	2	3	4	5	6	7
30	Seberapa puaskah Anda dengan pendapatan Anda? (1="sangat tidak puas", 7="sangat puas")	1	2	3	4	5	6	7

**Instruksi**<br>Lingkari jawaban yang paling sesuai dengan pendapat Anda. Tidak ada jawaban yang salah.								
**No**	**Pertanyaan**	**Jawaban**						
31	Seberapa puaskah Anda terhadap kondisi rumah tangga? (1="sangat tidak puas", 7="sangat puas")	1	2	3	4	5	6	7
32	Seberapa puaskah Anda terhadap rasa aman di lingkungan Anda? (1="sangat tidak puas", 7="sangat puas")	1	2	3	4	5	6	7
33	Seberapa puaskah Anda terhadap jaminan masa depan Anda? (1="sangat tidak puas", 7="sangat puas")	1	2	3	4	5	6	7
34	Seberapa puaskah Anda dengan pencapaian dalam hidup Anda? (1="sangat tidak puas", 7="sangat puas")	1	2	3	4	5	6	7
35	Seberapa puaskah Anda dengan ketersediaan waktu luang yang Anda miliki? (1="sangat tidak puas", 7="sangat puas")	1	2	3	4	5	6	7
36	Seberapa puaskah dengan situasi tempat tinggal Anda? (1="sangat tidak puas", 7="sangat puas")	1	2	3	4	5	6	7

Instruksi
Lingkari jawaban yang paling sesuai dengan pendapat Anda. Tidak ada jawaban yang salah.

No	Pertanyaan	Jawaban						
37	Seberapa puaskah Anda dengan kehidupan Anda secara keseluruhan? (1="sangat tidak puas", 7="sangat puas")	1	2	3	4	5	6	7
38	Apakah Anda merasa menjalani hidup yang bahagia? (1="tidak bahagia sama sekali", 7="sangat bahagia")	1	2	3	4	5	6	7
39	Apakah Anda merasa hidup Anda bermakna? (1="sama sekali tidak bermakna", and 7="sangat bermakna")	1	2	3	4	5	6	7
40	"Secara garis besar, hidup saya sudah sesuai harapan saya." (1="sangat tidak setuju", 7="sangat setuju")	1	2	3	4	5	6	7
41	"Situasi dalam hidup saya sangat baik." (1="sangat tidak setuju", 7="sangat setuju")	1	2	3	4	5	6	7
42	"Saya telah ,mendapatkan semua hal penting yang saya inginkan dalam hidup." (1="sangat tidak setuju", 7="sangat setuju")	1	2	3	4	5	6	7

**Instruksi**<br>Lingkari jawaban yang paling sesuai dengan pendapat Anda. Tidak ada jawaban yang salah.								
**No**	**Pertanyaan**	**Jawaban**						
43	"Apabila saya dapat mengulang kembali hidup saya, saya tidak akan merubah apapun." (1="sangat tidak setuju", 7="sangat setuju")	1	2	3	4	5	6	7

RESEARCH QUESTIONNAIRE		
**No**	**Question**	**Answer**
1	Birthday (dd/mm/yy)	…
2	Gender	a. Male b. Female
3	Birthplace (city)	…
4	Living area (SWK)	…
5	Highest formal education graduated from:	a. None b. Elementary school c. Middle school / equal d. High school / equal e. Academy / college / higher
6	Occupation	a. None b. Student c. Taking care of household d. Government employee e. Private sector employee f. Bussiness owner g. Army/ police officer h. Other
7	Marital status	a. Single b. Married c. Divorce/ widower
8	Total family income per month divided by the number of family members (IDR)	a. <1,554,360 b. 1,554,360 - 3,091,344 c. 3,091,345 - 5,000,000 d. 5,000,001 - 10,000,000 e. >10,000,000
9	How strong is your sense of belonging to Bandung city? (1= "do not have sense of belonging at all" and 7= "strong sense of belonging")	1 2 3 4 5 6 7

10	‘‘I feel proud of being Bandung's citizens’’ (1="strongly disagree", 7="strongly agreee")	1	2	3	4	5	6	7
11	‘‘Bandung is my home, no matter what’’ (1="strongly disagree", 7="strongly agreee")	1	2	3	4	5	6	7
12	How much trust do you have with Bandung's government? (1= “do not have trust at all” and 7= “completely trust”)	1	2	3	4	5	6	7
13	‘‘I am willing to pay more tax if that could improve social welfare’’ (1="strongly disagree", 7="strongly agreee")	1	2	3	4	5	6	7
14	‘‘I would like to spare part of my leisure time to do voluntary work.’’ (1="strongly disagree", 7="strongly agreee")	1	2	3	4	5	6	7
15	‘‘I trust my neighbors’’ (1="strongly disagree", 7="strongly agreee")	1	2	3	4	5	6	7
16	In social hierarchy, which social class do you belong to?	The lower class	The lower middle class		The middle class		The upper middle class	The upper class
17	"If someone is from a lower social class, I would avoid him or her." (1="strongly disagree", 7="strongly agreee")	1	2	3	4	5	6	7

18	"If someone is from a higher social class, I would avoid him or her." (1="strongly disagree", 7="strongly agreee")	1	2	3	4	5	6	7
19	"If someone has different political view, I would avould him or her." (1="strongly disagree", 7="strongly agreee")	1	2	3	4	5	6	7
20	"If my colleage has different religious view, I would avoid him or her." (1="strongly disagree", 7="strongly agreee")	1	2	3	4	5	6	7
21	"If someone is a migrant, I would avoid him or her." (1="strongly disagree", 7="strongly agreee")	1	2	3	4	5	6	7
22	"If someone lives on the government welfare system, I would avoid him or her." (1="strongly disagree", 7="strongly agreee")	1	2	3	4	5	6	7
23	How satisfied are you with your community? (1="not at all satisfied", 7="completely satisfied")	1	2	3	4	5	6	7
24	How satisfied are you with your social life? (1="not at all satisfied", 7="completely satisfied")	1	2	3	4	5	6	7

25	How satisfied are you with the harmony of your family? (1="not at all satisfied", 7="completely satisfied")	1	2	3	4	5	6	7
26	How satisfied are you with your personal relationships? (1="not at all satisfied", 7="completely satisfied")	1	2	3	4	5	6	7
27	How satisfied are you with your health? (1="not at all satisfied", 7="completely satisfied")	1	2	3	4	5	6	7
28	How satisfied are you with your education and skill? (1="not at all satisfied", 7="completely satisfied")	1	2	3	4	5	6	7
29	How satisfied are you with your job? (1="not at all satisfied", 7="completely satisfied")	1	2	3	4	5	6	7
30	How satisfied are you with your income? (1="not at all satisfied", 7="completely satisfied")	1	2	3	4	5	6	7
31	How satisfied are you with your housing situation? (1="not at all satisfied", 7="completely satisfied")	1	2	3	4	5	6	7
32	How satisfied are you with how safe you feel? (1="not at all satisfied", 7="completely satisfied")	1	2	3	4	5	6	7

33	How satisfied are you with your future security? (1="not at all satisfied", 7="completely satisfied")	1	2	3	4	5	6	7
34	How satisfied are you with what you are achieving in life? (1="not at all satisfied", 7="completely satisfied")	1	2	3	4	5	6	7
35	How satisfied are you with the leisure time that you have? (1="not at all satisfied", 7="completely satisfied")	1	2	3	4	5	6	7
36	How satisfied are you with your living situation? (1="not at all satisfied", 7="completely satisfied")	1	2	3	4	5	6	7
37	How satisfied are you with life as a whole? (1= "not at all satisfied" and 7= "completely satisfied")	1	2	3	4	5	6	7
38	Do you feel like you lived a happy life? (1="not happy at all", 7="completely happy")	1	2	3	4	5	6	7
39	To what extent do you feel the things you do in your life are worthwhile? (1="not at all worthwhile", and 7="completely worthwhile")	1	2	3	4	5	6	7
40	"In most ways, my life is close to my ideal." (1="strongly disagree", 7="strongly agreee")	1	2	3	4	5	6	7

41	"The conditions of my life are excellent." (1="strongly disagree", 7="strongly agreee")	1	2	3	4	5	6	7
42	"I have gotten the important things I want in life." (1="strongly disagree", 7="strongly agreee")	1	2	3	4	5	6	7
43	"If I could live my life over, I would change almost nothing." (1="strongly disagree", 7="strongly agreee")	1	2	3	4	5	6	7
